# The Chemistry of Reactive Oxygen Species (ROS) Revisited: Outlining Their Role in Biological Macromolecules (DNA, Lipids and Proteins) and Induced Pathologies

**DOI:** 10.3390/ijms22094642

**Published:** 2021-04-28

**Authors:** Celia Andrés Juan, José Manuel Pérez de la Lastra, Francisco J. Plou, Eduardo Pérez-Lebeña

**Affiliations:** 1 Cinquima Institute and Department of Organic Chemistry, Faculty of Sciences, Valladolid University, Paseo de Belén, 7, 47011 Valladolid, Spain; celia.andres.juan@uva.es; 2 Institute of Natural Products and Agrobiology, CSIC-Spanish Research Council, Avda. Astrofísico Fco. Sánchez, 38206 La Laguna, Spain; 3 Institute of Catalysis and Petrochemistry, CSIC-Spanish Research Council, 28049 Madrid, Spain; fplou@icp.csic.es; 4 Sistemas de Biotecnología y Recursos Naturales, 47625 Valladolid, Spain; info@glize.eu

**Keywords:** ROS, oxidative stress, macromolecules

## Abstract

Living species are continuously subjected to all extrinsic forms of reactive oxidants and others that are produced endogenously. There is extensive literature on the generation and effects of reactive oxygen species (ROS) in biological processes, both in terms of alteration and their role in cellular signaling and regulatory pathways. Cells produce ROS as a controlled physiological process, but increasing ROS becomes pathological and leads to oxidative stress and disease. The induction of oxidative stress is an imbalance between the production of radical species and the antioxidant defense systems, which can cause damage to cellular biomolecules, including lipids, proteins and DNA. Cellular and biochemical experiments have been complemented in various ways to explain the biological chemistry of ROS oxidants. However, it is often unclear how this translates into chemical reactions involving redox changes. This review addresses this question and includes a robust mechanistic explanation of the chemical reactions of ROS and oxidative stress.

## 1. Introduction

In chemistry, a free radical (FR) is a relatively stable species that contains one or more unpaired electrons and can react with other molecules, either by donating its unpaired electron to another molecule or by taking it away from another molecule to increase stability. In this way it converts the molecule with which it reacts into another FR, so a common feature of FR reactions is the chain process: one radical gives rise to another radical. This process only ceases when two FRs react with each other.

In a biological context, reactive oxygen species (ROS) are formed as a natural by-product of cellular aerobic metabolism. Mitochondrial respiration is a significant cause of reactive oxygen species (ROS) [1]. In addition to mitochondria, ROS are produced by a variety of enzymes such as NADPH oxidases (NOXs), xanthine oxidase, nitric oxide synthase, and peroxisomal constituents [2]. They are also produced by ionizing and UV radiation, as well as by the metabolism of a wide range of drugs and xenobiotics. In the endoplasmic reticulum, oxidants are released during the folding of proteins and the formation of disulphide bonds. They are highly reactive chemical molecules derived from the ability of the O_2_ molecule to accept electrons [3], generating subsequent unstable molecules such as superoxide anion (^•^O_2_^−^), hydrogen peroxide (H_2_O_2_), hydroxyl radical (OH^−^), and singlet oxygen (^1^O_2_^−^), produced by all kinds of cells. Stationary (physiological) levels of ROS are intrinsic to the normal functioning of cells, fulfilling the functions of cell signalling and homeostasis [4]. On the other hand, when they are produced in excess or when cellular defences are not able to metabolise them, oxidative stress (OS) damage occurs [5].

## 2. Genesis and Effects of ROS

**Figure 1:**
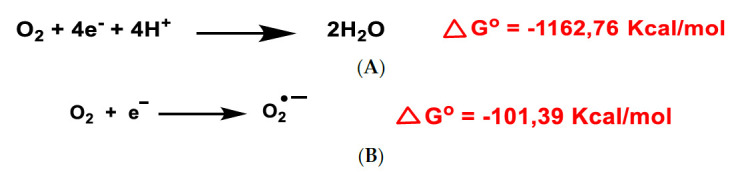
Gibbs free energy of O_2_ reduction yielding water (**A**) and the anion superoxide (**B**). Gibbs free energy calculated with Gaussian 09 software, revision D.01, method B3LYP/6-31G(d). Authors cited Note 1, after References.

All aerobic organisms need oxygen O_2_ for efficient energy production. Molecular O_2_ contains two unpaired electrons, but it is weakly reactive because they are located in different molecular orbitals and have a parallel spin. Consequently, oxygen preferentially accepts electrons one at a time. The ultimate goal of the electron transport chain ETC in the inner mitochondrial membrane (complexes I to V) is the reduction of the oxygen molecule to produce water. The first step of the O_2_ reduction reaction occurs spontaneously (Figure 1A).

**Figure 2:**
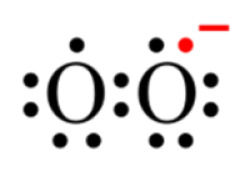
Lewis structure of the anion superoxide.

Univalent reduction of O_2_ gives rise to the anion superoxide (Figure 1B), which results from the addition of an electron filling one of the two uncompleted molecular orbitals, leaving a charged ionic species with a single unpaired electron and a net negative charge of −1 (Figure 2).

Superoxide ^•^O_2_^−^ is detrimental and is mainly produced as a by-product of mitochondrial respiration (especially in Complexes I and III, in the electron transport chain ETC) [6], where a small percentage of the electrons in the ETC chain escape from it, as well as by several other enzymes, which catalyse the electron transfer directly to molecular oxygen under strongly reducing conditions, as occurs in the mitochondrial matrix. It is also generated in the immune system to eliminate invading micro-organisms. In phagocytes, the enzyme NADPH oxidase produces ^•^O_2_^−^ in large quantities for use in the oxygen-dependent destruction mechanisms of invading pathogens [7].

**Figure 3:**
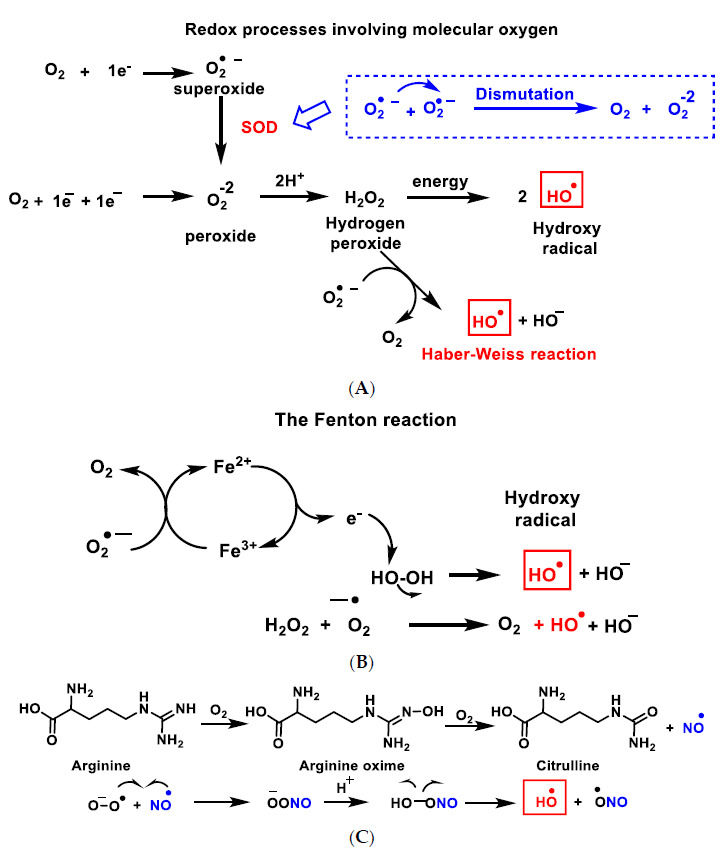
Reactive species ROS and RNS formed in the mitochondrial matrix by the Haber–Weiss reaction (**A**), the Fenton reaction (**B**) or by decomposition of peroxynitrite (**C**).

The hydroxyl radical ^•^OH (Figure 3A–C), the most powerful ROS oxidant, is formed during the Haber–Weiss reaction [8], by the Fenton reaction [9] or by decomposition of peroxynitrite [10], and has a very short half-life (10^−9^ s) and high reactivity.

O_2_ and ROS reduction are reactions in which the final product is water and have a negative Gibbs free energy, ∆G^o^ ≤ 0, so they occur spontaneously [11]. A small percentage of the electrons in the ETC transport chain, 0.1 to 2% of cases, pass through the chain and reach the mitochondrial matrix, where they prematurely reduce molecular oxygen O_2_ to superoxide ion ^•^O_2_^−^, the precursor of most reactive oxygen species.

## 3. Dual Role of ROS: Physiological and Pathological

ROS can be induced by exogenous sources such as tobacco, pollution, smoke, drugs, xenobiotics or ionising radiation, leading to irreversible effects on tissue development in animals and plants. In these, abiotic factors such as lack of water or high temperature can influence their emission. ROS play an important role in physio- and pathological processes. Mitochondria are particularly susceptible to oxidative damage, as electrons escaping from the ETC electron transport chain in the inner membrane react with oxygen to produce a superoxide anion. This anion is unstable and cannot cross membranes, but it is rapidly converted to hydrogen peroxide, which is permeable to the membrane. It can then undergo the Fenton reaction to produce the hydroxyl radical, which is highly reactive in the mitochondrial matrix. Elevated levels of ROS lead to increased mitochondrial DNA (mtDNA) damage.

In eukaryotic cells, ROS are mainly produced by biochemical reactions in mitochondrial cellular respiration processes (complex I and III, located in inner membrane). Transmembrane NADPH oxidases (NOXs) [12] and the mitochondrial electron transport chain (ETC) are the major endogenous enzymatic sources of ^•^O_2_^−^ and H_2_O_2_. However, they also come from sources such as peroxisomes, xanthine oxidase (XO), lipo- and cyclo-oxygenase and cytochrome P450 in endoplasmic reticulum [3]. In mitochondria, physiological levels of ^•^O_2_^−^ and H_2_O_2_ participate in redox signalling, but their production is significantly enhanced during oxidative stress conditions, producing an imbalance between ROS production and the action of the antioxidant defence system. They include SOD enzymes that reduce O_2_ into H_2_O_2_ (Zn-Cu SOD or SOD1 in cytosol and intermembrane space in mitochondria and Mn-SOD [13] or SOD2 in matrix mitochondria), catalase, glutathione peroxidases and thioredoxin reductase that convert levels of H_2_O_2_ into H_2_O and O_2_.

**Figure 4:**
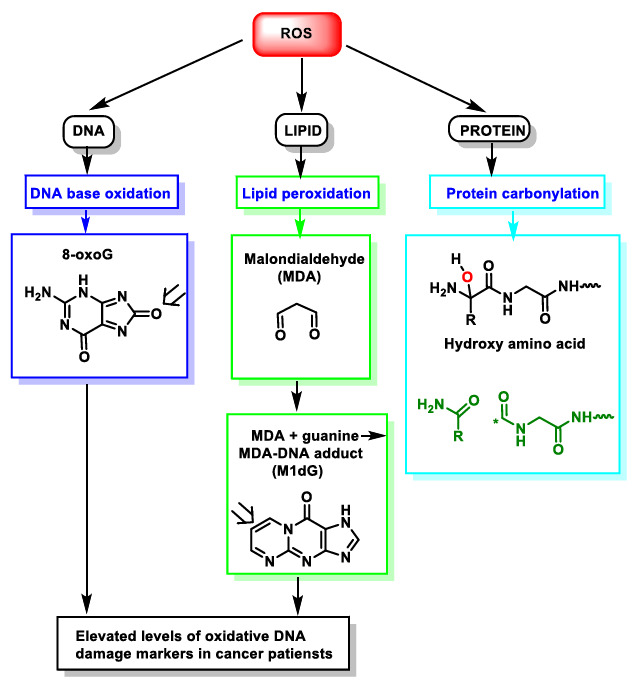
ROS action on DNA, lipids and proteins lead to DNA base oxidation, lipid peroxidation and protein carbonylation, respectively. * Unpaired electron.

During episodes of environmental stress, oxidative damage (OS) in cells can increase dramatically, causing damage to their structures [14]. Generally, the harmful effects of ROS in the cell include (i) damage on DNA or RNA [15]; (ii) lipid peroxidation of polyunsaturated fatty acids (such as membrane phospholipids) [16]; and (iii) oxidation of proteins [17], Figure 4. They cause irreversible damage to DNA, lipids and enzymes present in the cell cytosol, as they oxidise and modify cellular components and prevent them from carrying out their original functions.

## 4. ROS Damage on DNA

Nuclear and mtDNA have a separate evolutionary origin, as it is derived from the genomes of bacteria that were engulfed by the early ancestors of today’s eukaryotic cells. In extant organisms, most proteins present in mitochondria (in mammals, some 1500 different types) are encoded by nuclear DNA. mtDNA is particularly susceptible to reactive oxygen species generated by the respiratory chain [18], due to its proximity, despite being packaged with proteins as protective as those of nuclear chromatin. Its mutations can lead to a variety of diseases, as well as to the ageing process [19] and pathologies associated with advanced age. Evidence suggests a link between ageing and mitochondrial genome dysfunction [20].

**Figure 5:**
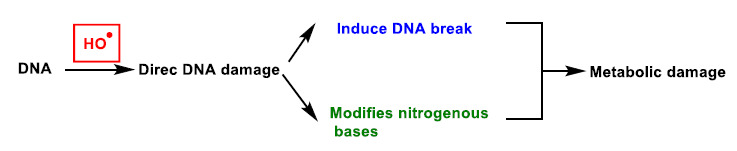
DNA damage caused by ROS.

DNA damage refers to physico-chemical changes in DNA [21], which can affect the interpretation and transmission of genetic information. It is damaged by a series of exogenous and endogenous stresses, which cause different forms of molecular modification (Figure 5). The DNA damage caused by ROS, from endogenous or exogenous sources, has been a significant breakthrough in carcinogenesis research over the last 20 years [22]. In DNA, ROS reacts with nitrogenous bases and deoxyribose, causing significant oxidative reactions. This can lead to mutations, carcinogenesis, apoptosis, necrosis and hereditary diseases. DNA fragmentation occurs, forced by the rupture of nucleosomes (fundamental structures for the organisation of DNA within chromosomes), thus causing problems in the compaction and coiling of DNA within chromatin. Chromatin plays an important role in the regulation of gene transcription [23], and thus alterations in its functional properties may result in errors leading to mutagenesis.

Oxidation of DNA can lead to alterations in DNA bases or double helix breaks, among other mutagenic alterations [24]. Hydroxyl radical stress causes direct damage to DNA, mainly by strand excision, and causes oxidative damage to the pyrimidine and purine bases. This process starts by radical-induced abstraction of a proton from any position of the deoxyribose and can result in many products.

**Figure 6:**
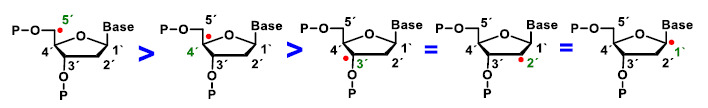
Abstraction of hydrogen at different carbons of deoxyribose by the ^•^OH radical. 5′H > 4′H > 3’H = 2´H = 1´H.

In deoxyribose, ^•^OH radical easily leads to hydrogen abstraction (also named hydrogen atom transfer HAT), forming different products and ribose fragments (Figure 6).

Several steps of the oxidation of deoxyribose at the C-4 position leads to a radical carbon stabilised by resonance with the oxygen in the ring. There is little experimental information available on which of the hydrogen atoms of DNA deoxyribose reacts with the hydroxyl radical. Experiments by Balasubramanian et al. provided a clear structural basis for rationalising the abstraction site preference. Indeed, the authors have clearly demonstrated the existence of a good correlation between the reactivity of the different sites and their solvent. This order of reactivity parallels the solvent exposure of the deoxyribose hydrogens [25].

**Figure 7:**
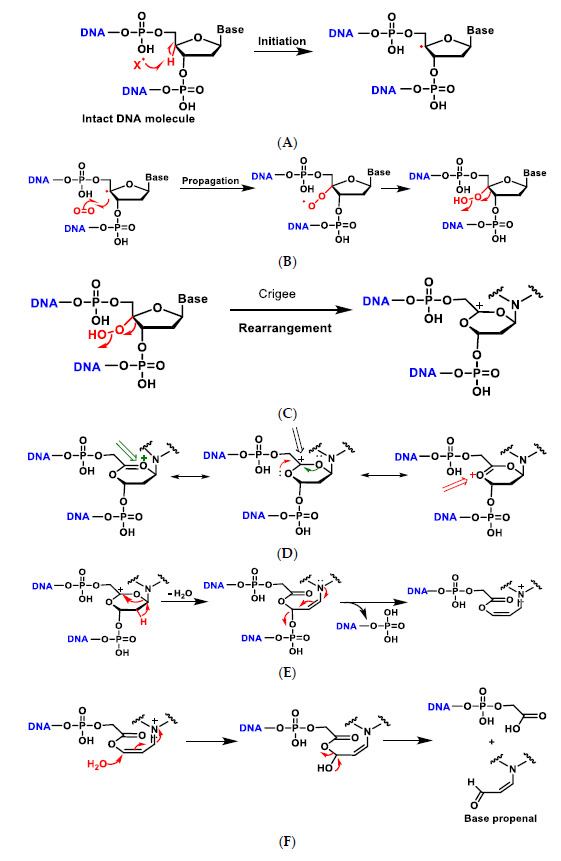

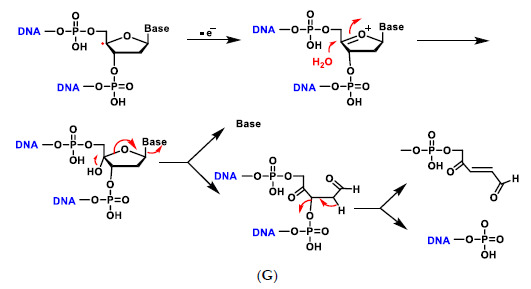
Mechanisms of oxidative damage to DNA-deoxyribose. The reaction with oxygen leads to several transposition reactions with expansion of the ring, which subsequently degrades to different products (**A**–**G**). (**A**) The reaction is initiated by abstraction of the hydrogen on the C-4 of deoxyribose by the hydroxyl radical or any other radical present in the medium to form the radical on the carbon; (**B**) The carbon radical reacts with O_2_ present in the reaction medium and transforms into the peroxyl radical which evolves into the hydroperoxide derivative; (**C**) The alkyl hydroperoxide formed undergoes a rearrangement, i.e., a migration from one atom or group of atoms to another within the same molecule, in this case with ring expansion to a six-linked ring and formation of the carbocation; (**D**) The generated carbocation is stabilised by delocalisation of the positive charge with the two adjacent oxygens and formation of the oxonium cation; (**E**) Dehydration with ring opening to form the enamine derivative which evolves to the unsaturated imine by loss of the phosphate residue; (**F**) Addition of water on the carbon and formation of the hydroxy acetal derivative that fragments to generate the acrylaldehyde-derived base; (**G**) In low oxygen environments the radical evolves to the oxonium cation and nucleophilic attack by a water molecule, then decomposes into the free base and the various fragments [26].

The addition of an O_2_ gives the peroxyl radical of the sugar, which is transformed into hydroperoxide and undergoes a transposition reaction with expansion of the ring, which subsequently degrades to different products such as enamine propenal derivatives (Figure 7A–G).

The mechanisms of oxidative damage to DNA bases involves abstraction and addition reactions by free radicals, with the formation of carbon-centred radicals. The hydroxyl radical causes direct DNA damage, by oxidation of pyrimidine and purine bases.

**Figure 8:**
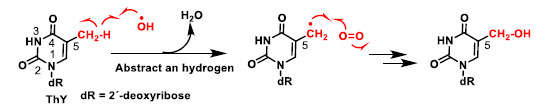
Abstraction of a hydrogen from the methyl group at position 5 by OH^–^ [27].

In thymine the abstraction of a methyl hydrogen from the 5-position by the hydroxyl radical generates a resonance-stabilised carbon radical (Figure 8), which provides the hydroxymethylene derivative, after treatment with oxygen and followed by reduction.

**Figure 9:**
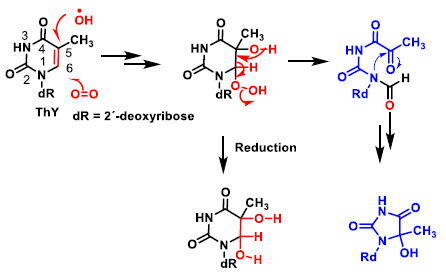
Reaction of the hydroxyl radical with pyrimidines is the double bond at the C5–C6 position.

The main site for the reaction of the hydroxyl radical with pyrimidines is the double bond at the C5–C6 position (Figure 9). Thymine after addition of the hydroxyl radical and reaction with oxygen is transformed into the hydroxy hydroperoxide derivative, which can be reduced to the diol or can undergo an opening process, as shown in the figure and subsequent cyclisation to the hydroxyhydantoin derivative.

**Figure 10:**
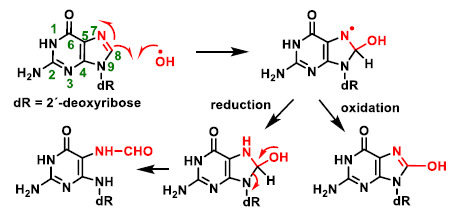
Hydroxylation of the C-8 position of the guanine derivative of DNA, generating degradation products [27].

Among other reactions, hydroxyl radicals can be added to the C-8 position of guanine and generate a radical on the nitrogen at position 7 (Figure 10), which can be reduced by the addition of an electron and a proton to an unstable intermediate, ultimately giving the ring-opening fragmentation product. Alternatively, it can undergo oxidation to give the derivative 8-hydroxyguanine. Hydroxylation of the C-8 position of the guanine derivative of DNA is the most well-studied DNA lesion induced by hydroxyl radical attack.

Mutations or deletions in the mitochondrial genome accumulate as cells mature, and are assumed to be triggered by long-term oxidative stress [28]. It has been shown that these mechanisms induce mtDNA disruption in neuronal degenerative disorders such as Alzheimer’s disease, Parkinson’s disease, and amyotrophic lateral sclerosis (ALS). Furthermore, these neuronal diseases facilitate the progression of additional DNA degradation during ageing, resulting in disease symptoms earlier than would be anticipated. This deterioration mechanism may result in more mtDNA mutation or depletion.

In addition to oxidizing DNA bases, ROS may cause DNA strand breakage due to free radical attacks on the DNA sugar-phosphate backbone [29]. DNA strand breaks may be identified as single-strand or double-strand breaks, and they can be mutagenic or clastogenic. A network of events known as the DNA damage response (DDR) is triggered in response to DNA damage [30]. DNA harm detection, checkpoint activation, cell cycle arrest, and finally repair, apoptosis, and immune clearance are all part of this reaction. Relevant pathways are triggered depending on the type of the DNA lesion to enable in the recognition of damaged regions and their repair.

Mitochondrial-based diseases are a group of disorders caused by their dysfunction and have unique characteristics because their function is critical to physiological cellular metabolism. In essence, mutations in mtDNA alter a careful balance between the production of reactive oxygen species (ROS) and their neutralisation by enzymes such as superoxide dismutase, catalase, glutathione peroxidase and others. Some mutations that increase ROS production ultimately induce oxidative damage to the mitochondrial matrix, inhibiting proteins such as Sirtuin3 (antioxidant and anti-tumour protein) [31] and reducing other redundant antioxidant defences, such as SOD2 [32].

Aging mitochondria are the critical factor in the origin of neurodegenerative diseases. In the brains of individuals with Alzheimer’s disease, there is high oxidative damage to nDNA and mtDNA, approximately 10-fold greater than in people without the disease. In Huntington’s disease, the mutant huntingtin protein causes mitochondrial dysfunction, with higher levels of ROS and increased oxidative stress [33].

During ageing, changes in mtDNA are well documented in the literature. Such changes include an accumulation of oxidised base pairs, base mismatches, strand breaks and deletions. Specific mutations and haplogroups of mtDNA emerge as predictors of both lifespan and risk of various age-associated diseases: cancer, diabetes, heart failure, sarcopenia and Alzheimer’s and Parkinson’s disease [34]. In neurodegenerative diseases, including amyotrophic lateral sclerosis (ALS), lipid peroxidation products and abnormal protein aggregation (the amyloid beta peptide) are biomarkers of oxidative stress [35].

**Figure 11:**
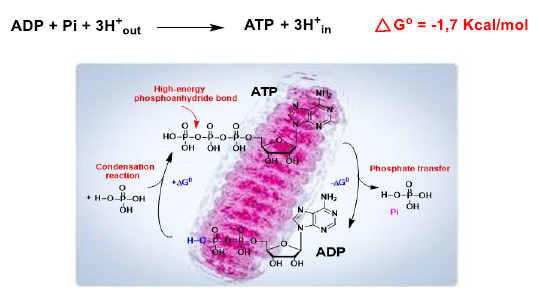
The ADP to ATP reaction in the mitochondria has a negative Gibbs free energy, ∆G ≤ 0, due to the reducing environment, and occurs spontaneously.

In mitochondria, H^+^ delivered to the intermembrane space is returned to the matrix during the oxidative phosphorylation reaction, so there is a reducing environment within the latter, facilitating the conversion of ADP into ATP (Figure 11).

Electrons from the ETC transport chain, in a small percentage of cases (0.1 to 2%), pass through the chain and reduce O2 in the mitochondrial matrix prematurely and incompletely to superoxide anion ^•^O_2_^−^, precursor of the reactive oxygen species [36].

Mitochondrial genome mutations accumulate in somatic tissues during normal ageing, as well as increased ROS production and biomarkers of ROS damage [37]. Demonstrating causality between the two has been difficult, as have direct links to ageing and longevity. The landscape of mitochondrial DNA mutations is open to spatiotemporal fluctuations throughout life, and it is likely that all individuals harbour mitochondrial DNA mutations, albeit at undetectable levels. Therefore, mitochondrial ROS production is highly dynamic and variable between cells [38].

## 5. Lipid Peroxidation Caused by ROS

Cell membranes are sensitive to radical damage due to the presence of polyunsaturated fatty acids. Another main effect of ROS is lipid peroxidation, which occurs when membrane phospholipids are brought into contact with an ROS oxidising agent. In this reaction, the free radical oxidises an unsaturated lipid chain, leading to the formation of a hydroperoxidised lipid and an alkyl radical. This lipoperoxidation results in alterations of the membrane structure, affecting its fluidity and damaging its integrity [39]. This process is initiated by the attack of a hydroxyl radical at one of the above-mentioned bis-allelic positions in the fatty acid side chains, leading to the generation of an alkyl radical.

The association of oxygen-derived free radicals with polyunsaturated fatty acids results in a number of extremely reactive electrophilic aldehydes during the process of lipid peroxidation. This effect happens as a result of continuing free radical chain reactions before they are terminated. Studies establishing a correlation between this type of oxidative disruption, neurodegeneration, and disease offered a wealth of information [40].

Peroxidation, which leads to atherosclerosis, is believed to occur inside blood vessel walls rather than (or to a lesser extent) in LDL circulating in the blood. Just as LDL can enter vessel walls, minimally modified LDL (LDL with some oxidation but not enough to be recognized by scavenger receptors) can escape back into the circulation. As a result, the sensitivity of circulating LDL to peroxidation can be a potentially valuable biomarker, suggestive of peroxidation in blood vessels [41].

**Figure 12:**
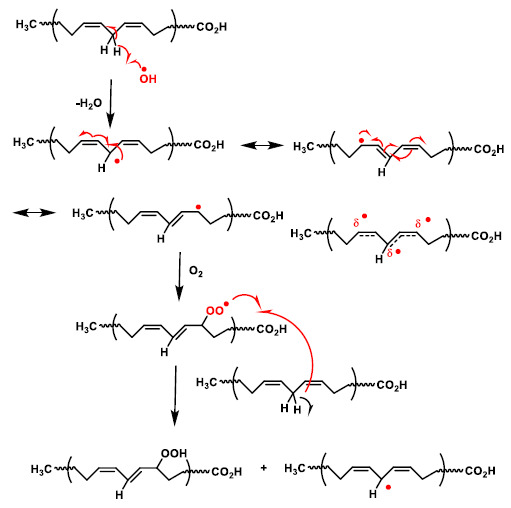
Mechanism of lipid peroxidation. The radical on the carbon reacts with an oxygen molecule to generate a peroxyl radical (R-O-O), which can abstract a new hydrogen atom from a double allylic C-H bond in the adjacent fatty acid side chain.

The initiation phase of lipid peroxidation (Figure 12). If a free radical attacks a carbon of the aliphatic chain of a fatty acid, hydrogen abstraction of the methylene group (-CH2-) attached to a carbon flanked by double bonds of a polyunsaturated fatty acid occurs, with the formation of a radical species. The radicals formed are stabilised by resonance with the double bond. In the propagation phase, a chain reaction occurs with the extension of the damage and the formation of further radical species. The radical formed in the first phase reacts with oxygen and forms a peroxyl radical (LOO), which can react with other adjacent polyunsaturated fatty acids to form a hydroperoxide and an alkyl radical, thus causing a chain reaction and damage to an increasing number of fatty acids.

**Figure 13:**
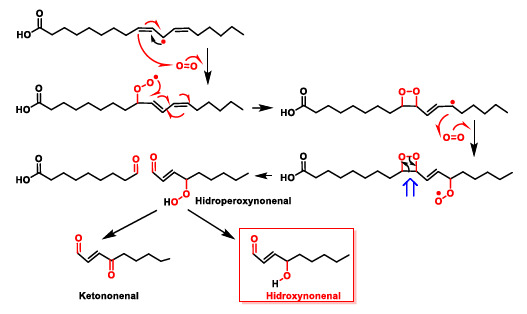
Toxic breakdown products from lipid peroxidation.

Lipid peroxidation can give rise to toxic breakdown products such as hydroxynonenal. Once formed, the peroxy radical cycles to a four-linked cyclic peroxide. The process increases with the number of unsaturations of the polyunsaturated fatty acid. Thus, when free radicals attack arachidonic acid (with four unsaturations) the initial abstraction of a hydrogen atom can occur at three points in the carbon chain of the fatty acid, because it has three methylene groups (-CH2-) attached to a carbon atom flanked by double bonds, thus increasing the complexity of the peroxidation reaction. The fatty acid undergoes a further reaction with oxygen followed by a break in the cycle to give hydroperoxynonenal, which is reduced to hydroxynonenal (Figure 13). Patients of Alzheimer’s disease have higher amounts of lipid-peroxidation compounds like 4-hydroxy-2-nonenal or acrolein in their brains [42], and elevated lipid peroxidation can also be seen in their cerebrospinal fluid and plasma. Increased oxidative stress and cell harm in this disease can be triggered by the association of transition metals, amyloid-peptide, and lipid peroxidation, according to recent studies.

**Figure 14:**
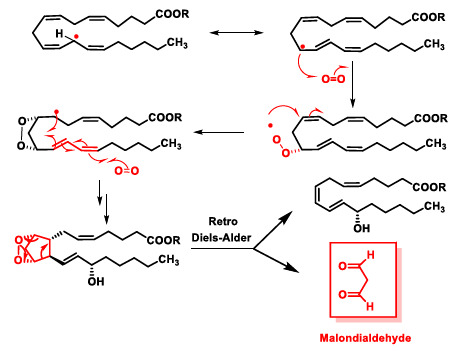
Lipid peroxidation of an arachidonic acid ester molecule.

Peroxidation of arachidonic acid leads to cyclic peroxides such as isoprostanoids in addition to hydroperoxides [43]. Alternatively, they can cycle together with the addition of a second oxygen molecule. These intermediates generate malondialdehyde (MDA) via a retro-Diels–Alder reaction. This MDA does react with DNA bases and causes mutagenic lesions (Figure 14).

**Figure 15:**
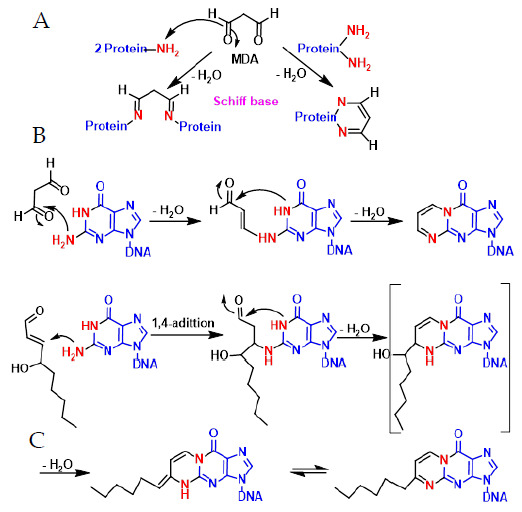
Mechanism of malonaldehyde reaction with proteins (**A**,**B**) and DNA (**C**).

In lipid peroxidation, toxic aldehydes such as malonaldehyde (Figure 15A–C) and hydroxynonenal are formed, which react with the -NH2 of proteins and DNA bases to form adducts that can cause mutations. Malondialdehyde MDA can covalently bind to amino groups of two different proteins, especially on lysine residues, or with two amino groups of the same protein and after water elimination forms imine derivatives or Schiff bases. It can also react with DNA bases and cause mutagenic lesions. MDA reacts with the amino group of guanine and after dehydration forms an enamine derivative, which eventually cycles to the six-linked ring after loss of a water molecule. With hydroxynonenal, the reaction is initiated by a 1,4 addition of the primary amino group of the guanine to the aldehyde-unsaturated position and subsequent formation of the imine and the cycle (Figure 15).

## 6. Protein and Enzyme Damage Caused by ROS

Proteins and enzymes are large, complex molecules that perform critical functions in the body. They are encoded by nuclear and mitochondrial DNA and do most of the work in cells. They are necessary for the structure, function and regulation of the body’s tissues and organs. Another critical aspect of OS is the damage triggered to the structural integrity of proteins, causing loss of catalytic activity of several enzymes and paralysis in the regulation of metabolic pathways. In the last 20 years, research linking lipid peroxidation and neurodegeneration has increased dramatically, particularly with the advent of proteomics, as each disease has become better understood. Not only in neurodegenerative diseases, but also in tumours, this recent area of research has given critical knowledge about protein modifications.

Unlike nucleic acids, oxidised proteins must be hydrolysed or processed by the proteasome to prevent their diffusion in the metabolic network or their interaction with other proteins. The effects of ROS on proteins are several: (i) oxidation of amino acid residues, (ii) cleavage of peptide bonds and (iii) aggregation between proteins. A wide range of diseases have been linked to the presence of oxidised proteins, such as Alzheimer’s disease, rheumatoid arthritis and others [44].

**Figure 16:**
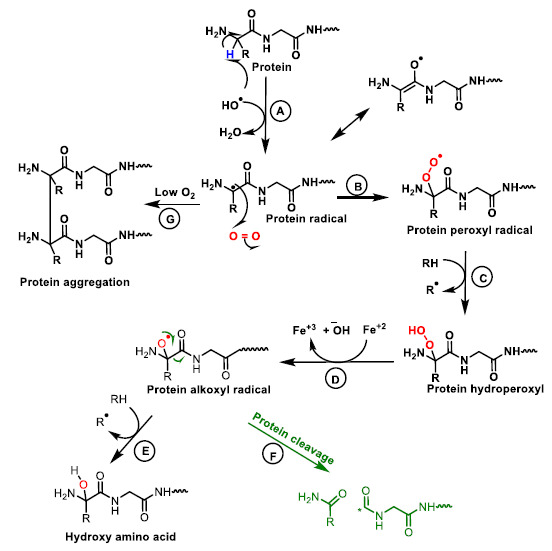
Mechanism of protein oxidation. The abstraction of hydrogen from the protein by the hydroxyl radical generates the alkyl radical, stabilised by resonance with the carboxyl function (**A**). The alkyl radical reacts with oxygen to form the peroxide radical (**B**). The peroxide radical abstracts another hydrogen from an adjacent protein and a hydroperoxide and an alkyl radical are formed (**C**). The hydroperoxide is reduced to an alkoxy radical in the presence of ferrous iron (**D**). Hydrogen abstraction from an adjacent protein by the alkoxyl radical forms hydroxy amino acid derivatives (**E**). The alkoxy radical upon cleavage generates different protein carboxy radicals and alkyl radicals (**F**). In the absence or at low oxygen levels the alkyl radicals form protein aggregates (**G**).

The nature of free radical-mediated protein damage depends on the amino acid composition (Figure 16).

## 7. Neutrophil Oxidative DNA Damage: Consequences for Human Health

ROS can pass through bacterial membranes and damage their nucleic acids, proteins and cell membranes. Consequently, bacterial pathogens employ various strategies to avoid the negative consequences of ROS production, such as directly preventing their secretion by using secreted effector proteins or toxins that interfere with the translocation of the NADPH oxidase complex, or even interfering with the signalling pathways required for their activation [45]. Several bacterial enzymes, such as superoxide dismutases (SOD), catalases and peroxiredoxins, are used to transform reactive species into products with lower toxicity. Catalases and peroxiredoxins act as H_2_O_2_ scavengers [46]. Free iron is necessary for the Fenton reaction to occur, so bacteria use a number of mechanisms to sequester it or to control its uptake in response to ROS in the environment [47]. DNA damage is a key consequence of ROS action and was thought to be the main mechanism of bacterial killing by ROS, particularly at the concentrations found in mammalian tissues. Oxidation of DNA bases by OH^−^ can produce several harmful by-products, and oxidation of ribose can induce strand breaks in bacterial DNA [48].

Neutrophils are normally found in the bloodstream (where they live for about a day), and in humans they constitute 40–70% of all white blood cells. They are formed from stem cells in the bone marrow and differentiate into neutrophil-killer and neutrophil-agent subpopulations, forming an essential part of the innate immune system. Neutrophils are short-lived and highly mobile, as they can be found in tissues where other cells are not. During the initial phase of a bacterial infection or in some cancers, neutrophils are one of the first cells to migrate to the site of inflammation, through blood vessels and then interstitial tissue, following chemical signals such as interleukin-8 (IL-8) and peroxide H_2_O_2_ in a process called chemotaxis [49].

Neutrophils are highly mobile and rapidly congregate at the focus of infection, attracted by cytokines expressed by activated endothelium, mast cells and macrophages. They recruit and activate other cells of the immune system, playing a key role in the first line of defence against invading pathogens, with three methods of attacking micro-organisms: phagocytosis (ingestion), degranulation (release of antimicrobials) and generation of neutrophil extracellular traps (NETs).

**Figure 17:**
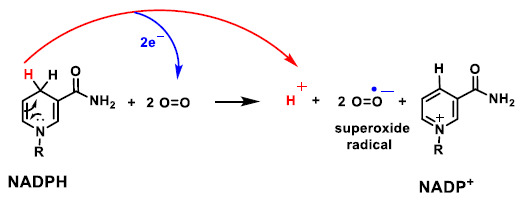
Mechanism of the reaction of NADPH with molecular oxygen and 2 electrons, producing NAPD+, the radical superoxide anion and one proton.

NADPH oxidase is an enzyme complex found in the cell membrane, in the outer space. It is found in the membranes of phagosomes used by neutrophilic white blood cells to engulf micro-organisms. This enzyme catalyses the production of a superoxide anion by transferring an electron to oxygen from NADPH (Figure 17).

ROS generated by NADPH oxidase play an important role in host antimicrobial defence and inflammation, activating animal immune responses and plant signalling. Superoxide anion directly kills bacteria and fungi, as the virulence of many pathogens is attenuated when their superoxide dismutase (SOD) genes are knocked out. In essence, insufficient activity can lead to increased susceptibility to microorganisms, but excessive action can lead to oxidative stress and cell damage. Careful regulation of NADPH oxidase activity is crucial for maintaining a healthy level of ROS in the body [50].

Vascular NADPH oxidases are regulated by a number of hormones and factors involved in vascular remodelling and disease, such as thrombin, platelet-derived growth factor (PDGF), tumour necrosis factor (TNF-α), lactosylceramide, IL-1 and oxidised LDL, by agonists and by arachidonic acid [51]. Mutations that alter the function of NADPH oxidase led to chronic granulomatous disease, characterised by severe infections and inflammatory disorders [52].

The formation of lipid hydroperoxides by neutrophil-derived ROS can also cause DNA damage. This process will produce a number of highly reactive side products, such as epoxides and aldehydes. Malondialdehyde and 4-hydroxynonenal, for example, have been widely researched and shown to be extremely DNA reactive and mutagenic [53].

Moreover, neutrophils play crucial roles in liver repair by promoting the phenotypic conversion of proinflammatory Ly6ChiCX3CR1lo monocytes/macrophages to proresolving Ly6CloCX3CR1hi macrophages. ROS expressed by neutrophils are important mediators that trigger this phenotypic conversion to promote liver repair. This conversion is prevented by neutrophil depletion through anti-Ly6G antibody, genetic deficiency of granulocyte colony-stimulating factor or genetic deficiency of NADPH oxidase 2 (Nox2) [54]. Adoptive transfer of WT neutrophils in place of Nox2-/rescues the altered phenotypic conversion of macrophages in neutrophil-depleted mice [55].

## 8. ROS-Mediated Activity of Antimicrobial Peptides

Antimicrobial peptides (AMPs) are mainly gene-encoded peptides, generally positively charged, with molecular weights less than 10 kDa. The majority of AMPs have small amino acid residue spans, ranging from 5 to 40, displaying diverse amino acid sequences and secondary structures forming amphipathic helixes. AMPs can bind to the microbial cell wall or membrane through electrostatic and hydrophobic interactions, disrupting the membrane [56]. AMPs are made with a marginal amount of energy and biomass, due to their limited molecular size, and are synthesized in a fast and versatile manner [56,57]. Unlike standard antibiotics, AMPs typically work on a large variety of pathogens, including parasites, enveloped viruses, fungi, gram negative and positive bacteria, and cancer cells [58].

Furthermore, unlike traditional antibiotics, which target a certain metabolic enzyme and can cause microbe resistance, AMPs destroy microorganisms primarily through a process requiring membrane destruction, which is normally difficult for microbes to overcome resistance [58]. The amino acid sequence as well as the molecular conformation determine the antimicrobial spectra of individual peptides. Several attempts have been made to establish AMPs as therapeutic agents, mainly for the treatment of external infections such as oral mucositis-related infections, chronic lung infection associated with cystic fibrosis, diabetic ulcers, ocular infections, and buco-dental infections; however, none have entered clinical application yet [58].

Recently, the antimicrobial activity of antimicrobial peptides has been related to the production of ROS. For example, PMAP-23, a member of the cathelicidin family in pigs, induces a Ca^2+^-dependent NADH oxidation that dramatically increased oxidized NADH levels, suggesting that elevated oxidation was induced by mitochondrial Ca^2+^ [59]. Reduced mitochondrial Ca^2+^ levels inhibited NADH oxidation, which is consistent with the assumption that NADH oxidation necessitates the entrance of Ca^2+^ ions into the mitochondria. These findings were consistent with PMAP-23 inducing a Ca^2+^-dependent NADH oxidation. Increased levels of ROS disrupt intracellular redox homeostasis decreasing GSH levels and changes the intracellular environment to a more oxidative state. PMAP-23 induced apoptosis in *C. albicans* cells via mitochondrial Ca^2+^-induced ROS. The overproduction of mitochondrial ROS compromised *C. albicans*’ intracellular redox homeostasis and decreased glutathione levels in fungal cells, resulting in oxidative stress [59].

Antimicrobial strategies such as ROS may be a feasible choice for coping with the increasing antimicrobial resistance situation. ROS have the potential to be a powerful weapon in the fight against microbial infections since they are toxic to a wide variety of pathogens by inducing oxidative stress [60]. ROS-induced oxidative stress may destroy cellular macromolecules like DNA, resulting in breakdown and, finally, microbial cell death. The potential application of ROS-based techniques as therapeutic therapies for microbial infections remains difficult, but at least some of them could become well known and adequately developed to enter clinical practice [59,60].

## 9. Oxidative Phosphorylation and Mitochondrial Uncoupling

Inside cell and mitochondria, the harmful effects of ROS include structural changes in DNA and RNA, peroxidation of membrane phospholipids (composed of polyunsaturated fatty acids) and oxidation of cellular proteins that perform numerous functions inside the cell. It is therefore important to keep ROS levels at physiological values, preventing them from increasing and triggering the expression of the mitochondrial protein UCP2 on its inner membrane [61]. The uncoupling protein (UCP) family acts as a proton channel or transporter, housed in the mitochondrial inner membrane. Therefore, it is able to dissipate the gradient from the mitochondrial matrix to the mitochondrial intermembrane space. The energy lost in dissipating the proton gradient generates heat, thus linking UCPs to thermogenesis.

UCPs are located in the same mitochondrial inner membrane as ATP synthase, which is also a proton channel. Both proteins work by introducing protons from the intermembrane space into the matrix, UCP2 generating heat and the other synthesizing adenosine triphosphate ATP from adenosine diphosphate ADP and inorganic phosphate, the last step of oxidative phosphorylation OSPHOS [62]. OXPHOS is based on the transport of H^+^ protons from the mitochondrial intermembrane space to its matrix, thus contributing to the reduction of molecular O_2_ to H_2_O. The disruption of this process is known as the Warburg effect [63].

Around 1920, Otto Heinrich Warburg and his group deduced that glucose and oxygen deprivation in tumour cells leads to lack of energy and ultimate apoptosis of the cells. Biochemist Herbert Grace Crabtree extended Warburg’s research by discovering environmental or genetic influences. Crabtree observed that *Saccharomyces cerevisiae* prefers glucose fermentation to ethanol over aerobic respiration in the presence of a high concentration of the former [64]. Warburg observed something similar in tumours: cancer cells tend to use glucose for energy even under aerobic conditions, coining the term “aerobic glycolysis”. It was hypothesized that dysfunctional mitochondria may be the cause of the higher rate of glycolysis observed in tumour cells, as well as a predominant cause of their development and proliferation [65]. This Warburg reprogramming occurs in many different types of cancer and is related to the Gordian knot in cancer treatment [66]. Cancer cells cause glycolysis to predominate over mitochondrial oxidative phosphorylation as a source of energy storage. Aerobic glycolysis with lactate generation is an adaptive strategy to the new metabolic needs of neoplastic cells and causes an increasingly aggressive cancer phenotype [67]. Thus, the origin of most cancer is now considered to be directly linked to mitochondrial dysfunction, which in turn is caused by ROS-induced oxidative mechanisms. There is therefore a direct link between ROS and cancer, which has also been observed in other diseases, such as Alzheimer’s and Type 2 diabetes [68].

**Figure 18:**
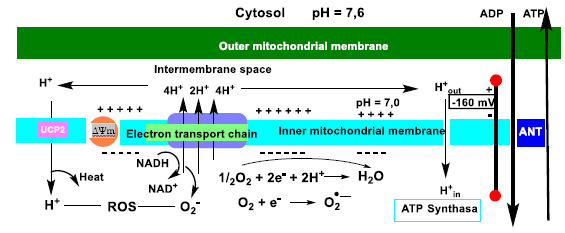
Schematic representation of oxidative phosphorylation and mitochondrial uncoupling. Electrons derived from glucose and fatty acid metabolism flow through complexes I-IV of the ETC electron transport chain in the mitochondrial inner membrane. The energy gradient of this process is used to pump protons (H^+^) from complexes I-IV to the intermembrane space. The resulting H^+^ gradient sustains the membrane potential ΔΨm, which drives ATP synthase (and subsequent oxidative phosphorylation). ATP and ADP are exchanged between the matrix and the cytoplasm via the adenine nucleotide translocase ANT. UCP2-induced proton uptake reduces ΔΨm values and a decrease in ATP production. This limitation of ΔΨm accelerates electron transport and mitochondrial respiration, limits the likelihood of electron leakage and the production of superoxide anion.

Mitochondrial respiration is coupled to ATP synthesis via this ADP phosphorylation step, i.e., protons introduced by ATP synthase are used to reduce molecular O_2_ to H_2_O. Activation of the UCP2 protein occurs by an increase in ROS emitted [69], thus providing an important mechanism for limiting the production of ROS, such as superoxide anion ^•^O_2_^−^, Figure 18.

In neoplastic cells, mitochondrial metabolism is modified to meet increased energy needs and the requirements associated with rapid and uncontrolled proliferation. One of the most prominent changes occurs in cellular energy metabolism, and is known as the.

An additional benefit of the Warburg effect is the detour of ETC substrates to decrease mitochondrial ROS production [70]. Cancer cells exhibit increased levels of intracellular ROS with complex biological effects. ROS induce genomic instability and stimulate cancer cell growth and survival by inhibiting their apoptosis. UCP2 expression is associated with cancer and modulates energy metabolism in response to elevated ROS levels [71]. Overexpression of UCP2 is found in leukaemia, ovarian, bladder, oesophageal, testicular, colorectal, kidney, pancreatic, lung and prostate tumours [72]. The effect of UCP2 on ATP concentrations varies according to cell type. The β-pancreatic cells undergo impaired ATP production with increased UCP2 activity, associating this process with cell degeneration, decreased insulin secretion and the onset of type II diabetes mellitus [73]. The increased number of mitochondria increases the combined concentration of ADP and ATP, resulting in an increase in ATP when the proton leakage mechanism is inhibited.

Zhang et al., 2006, demonstrate that, consistent with in vitro studies, overexpression of UCP2 leads to tumour development in vivo in an orthotopic model of breast cancer. Genipin, a phytochemical molecule, suppresses the tumorigenic properties of UCP2, mediated by downregulation of reactive oxygen species and downregulation of UCP2. This study demonstrates that: (i) the Warburg effect is mediated by UCP2; (ii) this protein is overexpressed in breast cancer and many other cancers; (iii) it promotes tumorigenic properties in vitro and in vivo; and (iv) genipin suppresses the tumour-promoting function of UCP2 [74].

## 10. ROS-Mediated Damage in Endoplasmic Reticulum (ER) and in Haemolytic Diseases

The endoplasmic reticulum (ER) is an organelle present in most eukaryotic cells, specialised in protein folding and transport. Alterations in the ER environment leads to the accumulation of misfolded proteins and this seriously affects various cell signalling processes, such as reduction-oxidation (redox) homeostasis, energy production, inflammation, differentiation and apoptosis. Reactive oxygen species (ROS) have been linked to cellular stress, and play a key role in many cellular processes occurring in the cytosol and in various organelles, such as the ER and mitochondria. Alteration of ROS homeostasis in the ER is sufficient to cause ER stress. It is also unclear how changes in the protein folding environment in the ER causes oxidative stress. Furthermore, it is unknown how ROS production and protein misfolding induce cell apoptosis and contribute to several degenerative diseases [75].

The unfolded protein response (UPR) is a group of adaptive signalling pathways whose function is to resolve protein misfolding and restore an efficient environment for protein folding. UPR signalling arises in response to protein misfolding in the ER. Prolonged ER stress leads to activation of the pro-apoptotic UPR, which plays a critical role in physiological and pathological conditions [76].

Haemolytic anaemias are caused by haemolysis, i.e., the abnormal breakdown of red blood cells (RBCs), either in the blood vessels (intravascular) or elsewhere in the human body (extravascular). During its course, red blood cells are destroyed faster than they can be made. Oxidative stress aggravates the symptoms of many diseases, including haemolytic anaemias, as it has been found in sickle cell anaemia and thalassaemia, glucose-6-phosphate dehydrogenase deficiency, hereditary spherocytosis, congenital dyserythropoietic anaemias and paroxysmal nocturnal haemoglobinuria. Although oxidative stress is not the origin of these diseases, OS of erythroid cells plays a crucial role in haemolysis due to ineffective erythropoiesis in the bone marrow and poor survival of red blood cells in the circulation. In addition, platelets and polymorphonuclear white blood cells (PMN) are also exposed to OS [77].

Correction of anaemia by red blood cell transfusions or iron supplementation may increase the oxidative stress burden. This situation suggests that both iron and redox status should be monitored during treatment, using red blood cells as biomarkers [78].

## 11. Conclusions

Oxidative stress is an early event in the aetiology of several diseases, as biomarkers of oxidative stress appear early in their development. The high chemical reactivity of ROS makes them very effective weapons against most biomolecules. Oxidants are implicated in an increasing number of varied and dynamic processes in molecular and cell biology research, where the underlying chemical pathways are often unknown. We here aimed to link chemistry and biology, and to put what could be considered a solid mechanistic foundation. We hope this review can help students and researchers to understand the mechanism of these highly reactive molecules and their role as mediators of oxidative modifications of cellular components.

## Data Availability

Not applicable.
